# Hand hygiene knowledge and demonstrated technique among Malawian kindergarten children: A quasi‐experimental study

**DOI:** 10.1002/nop2.1776

**Published:** 2023-04-25

**Authors:** Balwani Chingatichifwe Mbakaya, Maggie Zgambo, Fatch Welcome Kalembo

**Affiliations:** ^1^ Department of Public Health University of Livingstonia Mzuzu Malawi; ^2^ School of Nursing and Midwifery Edith Cowan University Perth Western Australia Australia; ^3^ School of Nursing Curtin University Perth Western Australia Australia

**Keywords:** 5‐step handwashing technique, hand hygiene, kindergarten, multi‐level approach

## Abstract

**Aim:**

To evaluate hand hygiene knowledge and demonstrated technique before and after implementation of the hand hygiene programme and its sustainability among Malawian kindergarten students.

**Design:**

Quasi‐experimental design, utilizing a repeated measure at three points, namely, before intervention (T_0_), soon after intervention (T_1_) and follow‐up (T_2_).

**Methods:**

The hand hygiene programme consisted of integrating hand hygiene protocol into the school health curriculum, setting up proper handwashing facilities, training school teachers, health talks and developing reminders on hand hygiene. Fifty‐three kindergarten children aged 3–6 years were enrolled in the programme. Data were collected at 3 months' intervals (T_0_, T_1_, and T_2_). Parents, teachers, school authorities and children were involved in the implementation and evaluation of the intervention, utilizing a multilevel approach.

**Results:**

There was a significant difference in knowledge scores across three time points (T_0_, T_1_ and T_2_), Chi‐Square (2, *n* = 53) = 79.02, *p* < 0.005 and handwashing technique across the three time points, Chi‐Square (2, *n* = 53) = 88.04, *p* < 0.005. There was a large effect size of 0.62 on the effect of handwashing technique scores from T_0_ to T_1._

## 1 BACKGROUND

Increased incidents of communicable diseases are often aggravated by poor hygiene practices and insufficient sanitary conditions (Freeman et al., 2017). Inappropriate and ineffective hand hygiene in kindergarten children are well documented to cause common infectious diseases such as diarrhoeal and respiratory infections, which are also the two most common causes of death in children (Mbakaya, 2022; Mbakaya et al., 2017; Suen & Cheung, 2020; Younie et al., 2020). Kindergarten children are at a higher risk for infection because of their immature immune systems, more frequent social mixing and underdeveloped fine motor skills to practice proper handwashing techniques (Suen & Cheung, 2020; Younie et al., 2020). Therefore, healthy lifestyle behaviours, such as proper handwashing techniques, must be introduced and promoted in the early developmental stage to enhance healthy lifestyle choices.

Globally, poor hand hygiene practices cause 443 million school days lost each year due to water‐related illnesses. Of these, 272 million are due to diarrhoea alone (World Vision International, 2019). Furthermore, in the school‐going population, poor hand hygiene practices cause 272 million days of school absenteeism each year globally (World Vision International, 2019). According to the 2018 report by the United Nations Children's Fund (UNICEF) and World Health Organisation (WHO), the availability of water and sanitation coverage in schools in developing countries is at 51%, compared to 89% in developed countries (UNICEF & WHO, 2018). These authors have also reported that only 21% of schools in developing countries have handwashing facilities and nearly two‐thirds of schools in developing countries have inadequate sanitation (UNICEF & WHO, 2018). While a lack of facilities for is problematic, the unavailability of soap for and the developmental stages of kindergarten children have also been documented as barriers to effective handwashing among young children (Luby et al., 2005). Young children do not often practice self‐hygiene or comprehend the importance of for behavioural change (Tengku Jamaluddin et al., 2020). In an attempt to improve handwashing in this group, researchers and implementers have utilized the antecedent and consequences approaches to teach hand hygiene to children. In the antecedent strategy, children are instructed to wash their hands, given rationales for every action in and given demonstrations to handwashing and prompted to wash hands (Au et al., 2010; Biezen et al., 2019; Jess et al., 2019; Jess & Dozier, 2020). In the consequences strategy, children are corrected for their shortfalls or reinforced for good behaviour with an incentive. However, Jess and Dozier (2020) in their brief review, reported that implementation of each of these strategies or components thereof is often not effective.

The WHO recommends a 7‐step technique to be utilised by the public (WHO, 2014). Recent studies have reported that this technique could be complex for young children considering the number of steps involved. It is possible that the complexity of the 7‐step procedure also contributes greatly to non‐compliance, in young children who often struggle to retain and remember information received in bulk (Lee et al., 2015; Lee & Lee, 2014; Mbakaya, 2022; Suen & Cheung, 2020). Following this, a simplified 5‐step handwashing technique was developed to reduce the number of steps in the procedure by two from WHO's 7‐step handwashing technique (Lee & Lee, 2014). “The simplified 5‐step technique combines steps 1 and 3, rubbing palms and fingers together (palm‐to‐palm and palm‐to‐palm with fingers interlaced steps), and omits the wrist‐rubbing procedure. The simplified 5‐step technique is as follows: (1) between fingers, (2) backs of hands, (3) backs of fingers, (4) fingertips, and (5) thumbs” (Lee & Lee, 2014). Developed in Hongkong, the 5‐step technique has been proven to reduce sickness‐related absenteeism for students with mild intellectual disability (MID) by 40 percent (Lee et al., 2022; Lee & Lee, 2014; Mbakaya, 2018).

While the 5‐step strategy is a promising strategy to improve in school‐going children, there is limited information regarding school‐going children and the use of a five‐step handwashing technique among kindergarten children globally. In Malawi, assessments regarding hand hygiene have focused on different populations (adults) (Rissman et al., 2020; Sheth et al., 2010; Slekiene & Mosler, 2020). Therefore, it is essential to evaluate hand hygiene knowledge and demonstrated technique before and after the implementation of a hand hygiene programme and its sustainability among kindergarten students in Malawi.

## 2 METHODOLOGY

### 2.1 Design

Quasi‐experimental study design from repeated measure at three time points, namely before – (T_0_), after – (T_1_) and follow‐up (T_2_).

### 2.2 Sample size

Based on the mean differences of 1.03 between pairs and the standard deviation of the differences of 0.74 as per findings from Lee and colleagues (2015) and assuming a power of 80% and a level of significance of 5% (two‐sided), a minimum sample size of 8 was needed (Dhand & Khatkar, 2014). Nonetheless, we recruited all pre‐primary children (53) who were enrolled at the school. All students were aged between 3 and 6 years and were in the same class.

### 2.3 Sampling technique

A convenience sample of all pre‐primary children (53) enrolled at a school in one of the cities in Malawi were approached to participate in the study. Informed written consent was obtained from their parents. All children were eligible to participate in the study as long as they were in a pre‐primary class, verbally provided assent and written parental consent, and did not have physical and mental challenges that could hinder them from developing knowledge and skills on hand hygiene. All children agreed to participate in the study. No child dropped out of the study.

### 2.4 Site

A private kindergarten school is situated in an urban setting in Malawi. The kindergarten is a nursery and reception school for a private primary school, which was one of the primary schools involved in a big cluster randomized controlled trial (RCT) study conducted in the 2016/2017 academic year. The trial was registered within ClinicalTrials.gov (https://clinicaltrials.gov), identifier number: NCT02968251. The purpose of the trial was to design and evaluate a school‐based handwashing programme in Malawi. Both the kindergarten and the primary schools were in the same compound brick fenced, separated by a brick wall and under the same directors. The two studies were conducted concurrently. The results of a big study are presented elsewhere (Mbakaya et al., 2019).

### 2.5 Intervention and fidelity test

The hand hygiene programme (HHP) was delivered for 30 min, once a week from 17th January 2017 to 5th May 2017. The intervention was implemented during this period because it is a wet season when Malawi experiences increased cases of waterborne diseases. The researchers wanted to promote hand hygiene among this group of children in order to reduce the incidences of waterborne diseases. The hand hygiene programme (HHP) had the following components: (1) integrating hand hygiene protocol into the school health curriculum. The school authority, head of school and all teachers agreed that the intervention be incorporated into the curriculum and be taught to students on daily basis during the period of the study; (2) setting up proper handwashing facilities by constructing handwashing sinks in consultation with the school authorities, teachers, parents and children; (3) training school teachers; (4) delivering health talks to kindergarten children on how, why and when to wash hands; and (5) developing reminders and posters of the simplified 5‐step handwashing technique and repeated demonstrations. Parents of the children were reached through a take‐home package, which comprised a simplified 5‐step handwashing poster, pamphlets and a commitment letter. Parents were also given details of the programme through an information sheet, which was given to each parent before they consented for their child to participate in the study.

The intervention was delivered by two competent and well‐trained qualified diploma Nurse Midwife Technicians (interventionists). The interventionists were given a one‐day training on how to implement the intervention by a public health specialist with a Master of Science in Public Health. The following topics were covered in the training: teaching of the simplified 5‐step handwashing techniques, demonstration of the simplified 5‐step handwashing techniques, and steps of implementing the intervention. The simplified 5‐step handwashing technique was adopted from a study conducted in Hong Kong (Lee et al., 2015). The competence of the two interventionists was assessed using a knowledge test and a 5‐step handwashing checklist to ensure that each one scored above 90%. The NMTs provided hand hygiene lessons to all students at the school. In addition, teachers for the reception class were invited to sit in the classroom when the NMTs were delivering hand hygiene to the children. Lesson plans were checked by the principal investigator prior to delivering each lesson. Both interventionists were available in the same class during lesson delivery and supplemented each other. Video clips were captured for sampled lessons and checked by the principal investigator.

The interventionists in collaboration with the teachers established two hand‐washing stations. Each station had a sink and a tap with running piped water supplied by Northern Region Water Board. The handwashing station also had a liquid handwashing soap, a single‐use paper towel for drying hands and a trash container. The interventionists demonstrated to children and teachers how to correctly watch their hands using the simplified 5‐step handwashing techniques at the handwashing station. The teachers and interventionists reminded the children about washing their hands several times a day (At the school morning assembly, before morning break and during lunch). The interventionists were available at the hand‐washing stations to support children with the correct hand‐washing techniques. Two other well‐trained diploma nurses not involved in the study assessed the students' knowledge and skill acquisition during the three time points. The assessment was done before programme implementation (T_0_), after programme implementation (T_1_) and 3 months after the completion of the intervention (T_2_). Although parents were expected to reinforce hand hygiene practices taught at school, their participation in the study was not evaluated. In a previous study (Lee et al., 2015), the inter‐rater reliability of the raters measured using intraclass correlation coefficients, was 0.89. In this study, the intraclass correlation coefficient was 0.949 (CI: 0.913: 0.970, *p*‐value = 0.000) showing very good agreement between the raters (strong correlation).

### 2.6 Study instruments and measurement

All three tools used to collect data in this study were already validated in previous studies. The first of the three study instruments used the demographic sheet, which was used to collect information on age, class and gender. Second, the handwashing quiz was used to collect information on students' knowledge regarding why and when to perform handwashing. The content was adapted from CDC (http://www.cdc.gov//when‐how‐handwashing.html). The instruments were validated and used in previous studies (Lee et al., 2015; Mbakaya et al., 2019). Furthermore, the tools were pre‐tested on 10 children in another school not involved in the study. Higher scores meant better knowledge and technique acquisition.

An instrument with questions and answers was used to assess handwashing knowledge gain. Marks were allocated to each question, and the children were awarded one (1) mark for a correct response. All wrong responses were graded as “0” (Appendix). At each time point, there were total knowledge scale scores (0–7).

Third, the adapted observational checklist was used to monitor the school children's competency in the adoption/acquisition of the simplified 5‐step handwashing technique (Kaewchana et al., 2012; WHO, 1996) (See Appendix). There were total scores for technique scales (0–8).

The scoring of the handwashing technique in this study was as follows: one mark was awarded for each of the following actions: use of soap, duration of handwashing (which is supposed to be more than 20 s), and proper air drying of the hands after washing or using a single use paper towel and throw in a trash container. These three accumulated a total of three marks, one for each action. The 5‐step handwashing technique was divided into 5 parts and accumulated a total of 5 marks. Rubbing between fingers scored 1 mark, rubbing the backs of both hands scored 1 mark, rubbing the back of one hand scored 0.5 marks, rubbing the backs of the fingers of both hands scored 1 mark while rubbing only one hand scored 0.5 marks. Rubbing the fingertips on the palm of both hands scored 1 mark (one hand only scored 0.5 marks). Rubbing the thumbs of both hands got 1 mark, while just one thumb scored only 0.5 marks. A total of 8 marks were possible for the simplified 5‐step handwashing technique.

### 2.7 Analysis

Raw data were entered in excel and analysed using Statistical Package for Social Science (SPSS) version 22. Descriptive statistics were generated to summarize the demographic characteristics of the study participants based on age and gender. Knowledge scores and skill acquisition (5‐step handwashing technique) of the participant before the programme, after the programme and follow‐up (sustainability test) were scored out of a total of 7, and 8 respectively at each point of measurement time. The quantified data were tested for normality using the Shapiro–Wilk test and were found to be non‐normally distributed (*p* < 0.05). Therefore, Friedman Test was carried out to determine the statistically significant difference in scores during the three time points (T_0_, T_1_ & T_2_) as in before‐programme, after‐programme and follow‐up.

Wilcoxon Signed Ranks Test was used to compare the differences in scores between before‐programme and after‐programme, and also between after‐programme and follow‐up in order to detect exactly where the differences in scores occurred as a post hoc test. The effect size was calculated by dividing the *Z*‐value of the Wilcoxon Signed Ranks Test by the square root of *N* (106). Cohen (1988) criteria of 0.1 = small effect, 0.3 = medium effect, and 0.5 = large effect were used to determine the impact of the programme.

### 2.8 Results

There were 53 children involved in the study, comprising 33 girls and 20 boys. There was no dropout during the entire study, with a 0% refusal rate. The mean age was 4.40 (0.768), with the youngest aged 3 years and the oldest aged 6 years. See Table 1.

**TABLE 1:** Demographic characteristics.

Variable	Frequency	Mean (SD)	Percent
Age in years			
3	6	4.40 (0.768)	11.3
4	23		43.4
5	21		39.6
6	3		5.7
Sex			
Girls	33		62.3
Boys	20		37.7
Total	53		100.0

### 2.9 Total scores on hand hygiene knowledge across time (T_0_ , T_1_ and T_2_ )

The results of the Friedman Test indicate that there was a statistically significant difference in the scores on hand hygiene knowledge across the three time points (T_0_, T_1_ and T_2_), Chi‐Square (2, *n* = 53) = 79.02, *p* < 0.005. Inspection of the median values showed an increase in the knowledge scores on hand hygiene from before‐programme (Median = 3.00: IQR = 2.00) to after‐programme (Median = 5.00: IQR = 2.00), and a further increase at follow‐up (Median = 6.00: IQR = 1.00). See Table 2.

**TABLE 2:** Scores on hand hygiene knowledge and 5‐step handwashing technique (*N* = 53).

	Before (T_0_)		After (T_1_)		Follow‐up (T_2_)	
	Median (IQR)	Mean rank	Median (IQR)	Mean rank	Median (IQR)	Mean rank
Knowledge	3.00 (2.00)	1.06	5.00 (2.00)	2.27	6.00 (1.00)	2.67
Technique	1.67 (1.59)	1.00	8.00 (0.67)	2.55	7.83 (0.92)	2.45

*Note*: Knowledge: Chi‐Square (2, *n* = 53) = 79.02, *p* < 0.005. Technique: Chi‐Square (2, *n* = 53) = 88.04, *p* < 0.005.

Abbreviation: IQR, inter‐quartile range.

### 2.10 Total scores on 5‐step handwashing technique across time (T_0_ , T_1_ , & T_2_ )

The results of the Friedman Test indicate that there was a statistically significant difference in the scores on the 5‐step handwashing technic across the three time points (T_0_, T_1_ & T_2_), Chi‐Square (2, *n* = 53) = 88.04, *p* < 0.005. Inspection of the median values showed an increase in the acquisition of the 5‐step handwashing technique from before‐programme (Md = 1.67: IQR = 1.59) to after‐programme (Md = 8.00: IQR = 0.67). However, there was a slight decrease in the 5‐step technic scores from after‐programme (Md = 8.00: IQR = 0.67) to follow‐up (Md = 7.83: IQR = 0.92). See Table 2.

### 2.11 Total knowledge scores between T_0_ and T_1_

A Wilcoxon Signed Rank Test revealed a statistically significant increase in the hand hygiene knowledge scores following the hand hygiene education programme, *Z* = −6.23, *p* < 0.001, with a large effect size of (*r* = 0.60). See Table 3.

**TABLE 3:** Knowledge and technique scores between T_0_ and T_1_; T_1_ and T_2_.

	Median difference	*Z*	*p*‐Value	ES(r)
Knowledge T_0_ and T_1_	2	−6.23	<0.005*	0.60
Knowledge T_1_ and T_2_	1	−3.48	0.001*	0.34
Technique T_0_ and T_1_	6.33	−6.34	<0.001*	0.62
Technique T_1_ and T_2_	−0.17	−0.77	0.44	0.07

*Note*: ∞ES(r): Cohen (1988) criteria of 0.1 = small effect, 0.3 = medium effect, 0.5 = large effect.

* *p*‐Value is significant at 0.05.

### 2.12 Total knowledge scores between T_1_ and T_2_

A Wilcoxon Signed Rank Test revealed a statistically significant increase in the hand hygiene knowledge scores following the hand hygiene education programme, *Z* = −3.48, *p* = 0.001, with a medium effect size of (*r* = 0.34). See Table 3.

### 2.13 Total 5‐step handwashing technique scores between time T_0_ and T_1_

A Wilcoxon Signed Rank Test revealed a statistically significant increase in the 5‐step handwashing technique scores following the hand hygiene programme, *Z* = −6.34, *p* < 0.001, with a large effect size of (*r* = 0.62). See Table 3.

### 2.14 Total 5‐step handwashing technique scores between time T_1_ and T_2_

A further analysis using Wilcoxon Signed Rank Test revealed a reduction in the skill acquisition of the 5‐step handwashing technique scores during the follow‐up, *Z* = − 0.77, *p* = 0.44. However, the reduction in the skills performance was not statistically significant (*p* > 0.05), with a small effect size of (*r* = 0.07). See Table 3.

## 3 DISCUSSION

To the best of our knowledge, this is the first study attempting to employ a multi‐level approach in which a non‐formal yet validated simplified 5‐step handwashing technique was used in an HHP among kindergarten children. The study found that there was a statistically significant difference in the scores on hand hygiene knowledge (*p* < 0.005) across the three time points (T_0_, T_1_ and T_2_). This shows that the implemented HHP was effective in increasing hand hygiene knowledge among kindergarten children. In addition, the children managed to retain the knowledge gained 3 months after the programme was stopped. This could be due to the involvement of the teachers during the programme implementation, and also parents through take‐home package. Songs were sung during the training session. In addition, a poster on proper handwashing including when, why and how to wash hand were put around the school to reinforce learning. The largest impact of the study was observed between T_0_ and T_1_ with a large effect size of 0.60 compared to sustainability follow‐up time (T_3_) which had a medium effect size of 0.34. However, in both situations, the effect of the HHP was statistically significant (*p* = 0.001). The findings of this study are similar to many studies which have demonstrated that knowledge level increases after the implementation of hand hygiene training (Lee et al., 2015; Mbakaya et al., 2017, 2019; Suen & Cheung, 2020; Younie et al., 2020).

The results of this study also indicate that there was a statistically significant difference in the scores on the 5‐step handwashing technique (*p* < 0.005) across the three time points (T_0_, T_1_ and T_2_), with a large effect size of 0.62 from T_0_ to T_1_. These findings are very important to the public health agenda and signify that investing in kindergarten children by equipping them with lifesaving skills is possible. This would help young children to form healthy habits early and use them throughout their life (Lee & Lee, 2014). Although there was a reduction in the 5‐step handwashing technique scores 3 between months after implementation of the programme (sustainability follow‐up; T_2_), the reduction was not statistically significant (*p* = 0.44) and the scores were still higher compared to before (T_0_) programme. A possible explanation could be the lack of frequent reminders by the interventionist which acted as motivators after the interventionist stopped. Furthermore, the children may have started forgetting the technique as time passed due to lack of practice.

Our study involved 53 younger children with a mean age of 4; SD = 0.77, compared to a similar study that used a simplified handwashing technique conducted in Hong Kong, in which the mean age was 10.55; SD = 2.61 (Lee et al., 2015). This is because, in this study, participants were kindergarten children, while in the Hong Kong study, they used school children. Kindergarten is the most vulnerable group of children who are yet to fully develop lifesaving skills such as a systematic handwashing technique. This group lacks fully developed fine motor skills (Suen & Cheung, 2020). Therefore, it is essential to invest in this age group in order to prevent respiratory infections and diarrhoeal disease which can be fatal and are predominantly transmitted through contaminated hands. These children stay in relatively clean homes and get exposed to other children who may have the infection in a relatively congested school setting.

The improvement in both hand hygiene knowledge and handwashing skills could be ascribed to the multi‐level approach which was used in this study to influence the children's hand hygiene behaviour at three different levels. An intervention is multilevel if it addresses the individual client and at least two levels of contextual influence, thereby targeting at least three different sources of influence (Taplin et al., 2012). For example, our study involved the child, their teachers, and their parents using different media. Multilevel intervention is believed to influence interdependent interaction, thereby producing desirable outcomes (Taplin et al., 2012).

The findings of this study, therefore, provide evidence that an HHP incorporating a simplified 5‐step handwashing technique in the context of a multi‐level approach is useful in promoting the acquisition of handwashing techniques, especially among vulnerable populations such as kindergarten children. The call for standardization of a simplified 5‐step handwashing technique was also made in a study conducted in Hong Kong among children with mild intellectual disabilities (Lee et al., 2015). Young children learn better through short and repeated instruction as is the case with five steps of handwashing compared to long official handwashing procedures which are recommended but not adhered to even by professionally well‐trained health workers. The simplified 5‐step handwashing technique is simpler and easier to memorise for kindergarten children whose cognitive and motor skills are still developing as evident in this study. These findings are in agreement with the findings of other studies conducted in Hong Kong, in which the authors found that the simplified 5‐steps technique of handwashing was easier to master and memorise than the 7‐step handwashing technique for children with mild intellectual disability (Lee et al., 2015; Lee & Lee, 2014). Children in kindergarten school are still young and immature socially, physiologically, psychologically and intellectually, as such, they take longer to follow instructions and master complicated procedures, and at the same time are more vulnerable to infections (Skinner, 2018). Therefore, the findings of this study capacitate policy makers to consider recommending the use of the simplified 5‐step handwashing technique in young children especially in the school setting, especially now that COVID‐19 is added to the list of infectious diseases which requires handwashing as one of the recommended preventive measures by the World Health Organisation. School and community health nurses can capitalise on the findings of this research to promote hand hygiene among school children and the community in general. Policy makers should also consider recommending teachers who spend most of their time working with students, to be trained to support the hand hygiene initiative in schools.

### 3.1 Limitations of the study

There are four limitations to our study. First, due to the nature of the design used, it was not possible to blind the interventionist or the enumerator. This could have introduced observer bias into the study. However, the interventionist and the enumerators were trained to adhere strictly to protocols. Second, we conducted this study in one private kindergarten school in one region of the country in the northern part of Malawi, and thus, the results may not be generalizable to other settings. Third, due to the small sample size used and the age of the data, the results of this study should be interpreted with caution. Future longitudinal studies should rigorously assess the effectiveness of this intervention in reducing incidences of respiratory and diarrhoeal infections among kindergarten children. Furthermore, future studies could replicate the current study by including kindergarten schools from all three regions of the country. As there was a fall off in the skills between T1 and T2, we suggest that researchers in future studies should consider including an additional follow up period (another 3–6 months).

## 4 CONCLUSION

A hand hygiene programme (HHP) for kindergarten children increased their hand hygiene knowledge and skill acquisition of the simplified 5‐step handwashing technique. It is paramount to standardize a handwashing programme for everyone who is directly or indirectly involved in the upbringing of young children, especially those in kindergarten schools, school and community health nurses in order to prevent infections in this vulnerable group. Hand hygiene needs to be emphasised especially during the COVID‐19 outbreak where is one of the best preventive measures. It is, therefore, expected that an HHP using a simplified 5‐step handwashing technique could be considered and promoted by the ministries of Health and Education through conducting training for community health workers, nurses, and teachers, who will, in turn, impart the skills to young children.

## AUTHOR CONTRIBUTIONS

All the authors contributed equally towards study conception, study design, data collection, analysis, interpretation and manuscript preparation. All authors read and approved the manuscript.

## FUNDING INFORMATION

There was no funding for this study.

## CONFLICT OF INTEREST STATEMENT

All authors declare no competing and conflict of interests.

## PATIENT CONSENT STATEMENT

Parents to all 53 children allowed their ward to participate and signed.

## RESEARCH ETHICS COMMITTEE APPROVAL

The study was ethically approved by a Research Ethics Committee (REDACTED) with a protocol number (REDACTED), as part of a big randomised controlled study conducted among private primary schools. Clearance was obtained from school authorities. Parents consented in writing for their wards to participate.

## Data Availability

Data available on request from the authors.

## APPENDIX 1 Scale for scoring simplified 5‐step hand washing technique.

### 1.1

**Table:**

Step	Left hand	Right hand	Total	Awarded mark
Rub palm to palm between fingers	_	_	1	
Rub back of hand	0.5	0.5	1	
Rub back of fingers	0.5	0.5	1	
Rub fingers on palm	0.5	0.5	1	
Rub the thumb	0.5	0.5	1	
Use of soap	_	_	1	
Duration of rubbing (>20 s)	_	_	1	
Proper air drying	_	_	1	
*Total marks*	*_*	*_*	*8*	
*Total %*	*_*	*_*	*100%*	

*Source*: Adapted from (Kaewchana et al., 2012; WHO, 1996).
